# Pharmaceutical indication pictograms for low literacy viewers: Health literacy and comprehension

**DOI:** 10.4102/hsag.v28i0.2192

**Published:** 2023-10-12

**Authors:** Ros Dowse, Sam Okeyo, Simise Sikhondze, Nosihle Khumalo

**Affiliations:** 1 Department of Pharmacy, Faculty of Pharmacy, Rhodes University, Makhanda, South Africa

**Keywords:** pharmaceutical pictogram comprehension, low health literacy, limited English, health communication, visual literacy, medicine packaging

## Abstract

**Background:**

Poor comprehension of pharmaceutical pictograms used on medicine labels or leaflets can compromise understanding of medicine-taking information, potentially causing negative health outcomes.

**Aim:**

The aim was to assess association of health literacy (HL) with comprehension of pictograms displaying indication and side effect information in a lower literacy, limited English proficiency (LEP) population.

**Setting:**

Community centre, Makhanda, South Africa.

**Methods:**

This was a quantitative cross-sectional study using simple random probability sampling. Ninety isiXhosa-speaking adults with a maximum of 12 years schooling, attending primary healthcare clinics were interviewed using structured interviews. Health literacy was assessed using the Health Literacy Test for Limited Literacy populations. Comprehension of 10 locally developed pictograms was evaluated.

**Results:**

The mean pictogram comprehension score was 7.9/10, with 8/10 pictograms complying with the International Organization for Standardization criterion of 66.7% correct comprehension. Only 15.6% of participants had adequate HL. A significant association of HL with pictogram comprehension was established (*p* = 0.002). Pictogram misinterpretation was higher in those with lower HL; adequate HL was associated with superior comprehension. Pictogram comprehension was negatively associated with age (*p* < 0.006), and positively associated with education (*p* < 0.001) and English proficiency (*p* < 0.001).

**Conclusion:**

Higher HL was associated with better pictogram comprehension. Low HL, LEP and low education levels are regarded as potential indicators for possible pictogram misinterpretation.

**Contribution:**

This study observed the potential for misinterpretation of medication pictograms. Health professionals should be aware that low HL, limited schooling and limited English proficiency could signal difficulty in fully comprehending pictogram content.

## Introduction

The communication of medicine-related information to patients should ensure good comprehension and retention of information as well as the ability to recall the information in order to support sustained, safe medicine-taking practice, particularly medicine adherence. This information is usually communicated verbally by health professionals. However, research has found that words are not the most effective mode of communicating health and medicines information to patients. Between 40% and 80% of the communicated information is forgotten immediately, with up to half of the information incorrect (Kessels 2003). Although medicine information is usually available as a leaflet inside medicine packaging and may also be accessible online, the readability level is often reported as being inappropriately high, of poor quality and does not align with patients’ needs (Jairoun et al. 2022; Van Beusekom et al. 2016). This particularly affects the more vulnerable patient groups such as those with lower literacy skills, limited English proficiency (LEP) and the elderly, as well as many immigrant and refugee populations (Dowse, Ramela & Browne 2011; Nualdaisri, Corlett & Krska 2021).

Many patients, particularly in low- and middle-income countries (LMICs), are likely to have limited literacy skills and inadequate health literacy (HL) and typically receive no written information. Low HL and LEP have been identified as barriers to health communication. These constructs are associated with poorer self-reported health status, increased medicine-taking errors and reduced medication adherence, resulting in negative health outcomes (Al Shamsi et al. 2020; Sentell & Braun 2012). These patients have greater difficulty in accessing and comprehending information relating to correct medicine usage instructions, indication and side effect information, and risks and warnings (Wolf et al. 2006).

Pictograms are the most commonly used pictorials in the health literature. They can be defined as stylized, figurative, two-dimensional images intended to attract attention and convey information without language or words (Abdullah, Hübner & Cziwerny 2006). The inclusion of pictograms on medicine labels and in written health information leaflets has been shown to improve comprehension, knowledge and recall of information (Dowse & Ehlers 2005; Heyns, Van Huyssteen & Bheekie 2021; Merks et al. 2018; Mohammad et al. 2022). They also serve to attract the viewer’s attention, enhance user-friendliness and reduce the perceived challenges associated with reading text-only documents, and they improve patient satisfaction (Mansoor & Dowse 2003). Studies have reported improved recall of information when verbally communicated instructions are enhanced by the concomitant use of pictograms (Dowse et al. 2011; Wilby et al. 2011).

Health literacy interventions aimed at improving comprehension have included the incorporation of visuals or pictograms. This has been informed by reviews of the literature, which support the use of pictorial health information, reporting that it increases knowledge and understanding for patients and consumers, particularly for individuals with lower HL (Mbanda et al. 2021). A 2020 systematic review aimed solely at investigating the effect of pictograms on medication adherence found that 10 of the 17 included intervention studies reported a significant positive effect of pictograms on medication adherence when used in combination with oral counselling or text-based medication information (Sletvold, Sagmo & Torheim 2020).

The successful interpretation of a pictogram and its elements requires visual literacy. This can be described as the abilities to understand (or read), and use (or write) images in order to communicate information to others (Avgerinou & Pettersson 2011). Unfortunately, the ability to develop visual literacy in terms of learning how to ‘read’ visuals is not actively taught in the school curriculum; most viewers develop this skill informally by repeated exposure to visuals that are now an integral part of our daily lives.

Pictograms are two-dimensional static visuals that are limited in what they can convey. They often demand the use of graphic conventions (e.g. an arrow indicating movement in a certain direction), which require active learning. Pictograms do not constitute a universal language as even a well-designed and easily comprehended pictogram in one group may be poorly comprehended by a different group. Pictograms are open to misinterpretation and confusion (Cloutier et al. 2014; Dowse & Ehlers 2004), particularly when used as the sole mode of communication without supporting verbal or written information (Mansoor & Dowse 2003; Van Beusekom et al. 2016; Wolf et al. 2006). Unintentional errors in medicine usage because of pictogram misinterpretation could then be associated with negative health outcomes. Factors that may predispose pictograms to misinterpretation include images that are culturally inappropriate or unfamiliar, have high complexity that imposes a high cognitive load on the viewer, poor legibility of elements comprising the visual and poor pictogram design (Dowse et al. 2010; Lühnen, Steckelberg & Buhse 2018; Van Beusekom et al. 2017).

The aim of this study was to assess the association of HL with comprehension of pharmaceutical pictograms displaying indication and side effect information in a lower literacy, LEP population.

## Research methods and design

### Source of pictograms and their modification process

As part of an overarching study, 10 pictograms designed at Rhodes University had been identified for modifying and testing. These pictograms illustrated a range of indications and side effects: general body pain, constipation, diarrhoea, cough, dizziness, headache, heartburn, rash, fever and vomiting. Six pictograms designed and tested in a 2009 study were included in an illustrated information leaflet for low-literate HIV patients on antiretroviral therapy (Dowse et al. 2011; Ramela 2009). Following an external request in 2010, a further four pictograms were developed by the first author (R.D.) in collaboration with members of the same target population and our graphic artist. In a 2012 undergraduate research study, nine of the current study pictograms were assessed for comprehension in 40 isiXhosa participants, but with different inclusion criteria relating to education level (unpublished data).

The International Organization for Standardization (ISO) criterion requires correct comprehension of symbols by at least 67% of users (ISO 9186 1:2014 Graphical Symbols, 2014). The 2012 study identified that only 3 of the 9 pictograms (headache, rash and vomiting) complied with this criterion, along with the single included pictogram from the 2009 study (Ramela 2009). In attempting to comply with the ISO criterion, modification of the 10 current study pictograms had therefore aimed to improve visual clarity, legibility and overall interpretive complexity in order to reduce the cognitive load on the viewer and to enhance comprehension of all pictograms. The methodology of the first phase of this overall project, which involved the detailed modification process of study pictograms, has been reported elsewhere (Dowse et al. 2022).

### Study design, setting and population

This was a cross-sectional quantitative study design using simple random probability sampling, with structured interviews used for collecting data. The research setting was at Makhanda, Eastern Cape, a poor, largely rural province of South Africa. Interviews were conducted in a local community development centre. Most people living in this area are of low socioeconomic status (SES). Participant inclusion criteria included being first-language isiXhosa, attendees of public primary healthcare clinics, at least 18 years old and a maximum of 12 years of schooling with no post-school courses. Participants were stratified into two schooling categories: 0–7 years and 8–12 years.

### Questionnaire, recruitment and data collection

A questionnaire was developed to facilitate collection of the following data: participant demographics, digital access and use, comprehension of pictograms and acceptability of pictograms and their use. Health literacy was assessed using the Health Literacy Test – Limited Literacy (HELT-LL)developed and validated in South Africa (Marimwe & Dowse 2019). Participants were recruited from the area surrounding a local community development centre. An A5 recruitment flyer was developed for distributing and posting at strategic local venues. A long-term employee at the development centre who was well known in the community assisted with the recruitment process. Sample size was determined using the Z-test for proportions of the power calculation. With a significance level of 5% and power of 80% to detect a predicted difference of 30% between two education groups (1–7 years and 8–12 years of schooling), the calculated sample size was 40 for each group. Ninety participants were recruited, with each student researcher conducting 30 interviews.

Data collection was performed in July 2019 – August 2019 at the community development centre. Individual interviews were conducted based on the structured questionnaire. The three student researchers individually conducted pilot interviews under the supervision of the first author who also sat in as an observer in the first three interviews of each student researcher. An interpreter who had been trained by the first author for prior research projects was present at all interviews to assist when necessary, as not all student researchers were fluent in isiXhosa. The interpreter ensured that study information and consent form content were clearly understood. All questions, apart from those in the HELT-LL, which were already translated into isiXhosa, were translated and communicated by the interpreter. All participants received an information letter and signed a consent form.

Prior to displaying the study pictograms to each participant, a sample pictogram was presented showing a man clutching his stomach in a forward leaning posture to reflect stomach-ache; participants were asked what they thought it illustrated. This was performed to introduce participants to the concept of ‘reading’ the visual elements in each pictogram and then assisting them in integrating the elements to derive the meaning of the overall visual. The 10 pictograms, printed on 11 cm × 9 cm cards, were then shown in random order, which was ensured by shuffling the pictogram pack between each interview. This is intended to avoid a familiarity bias effect, where later shown ones are often more easily comprehended as the participant becomes increasingly comfortable with the process of ‘reading’ and decoding the images (Shen, Xue & Wang 2018). For pictogram evaluation, the question ‘what do you think this pictogram means?’ was asked and the final response was noticed. Pictogram comprehension was scored as either incorrect (0) or correct (1). An overall pictogram interpretation score for each participant was generated by summating the correct answers.

Any comments or misinterpretations were documented. Potential uncertainties in point allocation were observed and these were later discussed and resolved in a joint meeting of the four researchers. Lastly, participants were asked whether they liked the pictograms and would want to see them used on their medicines. Patients were thanked and remunerated for their time and contribution to the study.

### Ethical considerations

Ethics approval for the study was obtained from the Rhodes University Ethical Standards Committee (0522-409). Participants received all information about the study both verbally and in writing. Participation in the study was voluntary, with no coercion. The study offered no physical, social or psychological risks. No identifying information was used on the questionnaires and total anonymity for all participants was ensured. Online information including data analysis spreadsheets and other study-related documents will remain in a password protected folder on the researcher’s computer for a period of 5 years.

### Data analysis

Data were analysed using Stata Statistical Software: Release 15. Frequencies were generated for all variables. Health literacy scores for each question ranged between 0 and 2, with the total maximum score being 24. Responses were categorised into three categories: adequate (18–24), marginal (11–17) and inadequate (≤ 10). The association of selected variables (age, education, English proficiency, HL) with pictogram comprehension was investigated using Chi-squared tests and ANOVA. Linear regression was used for correlating HL scores with pictogram comprehension scores. Fisher’s exact test investigated the association of each of the 10 pictograms with HL. Significance was set at *p* < 0.05.

## Results

A total of 90 participants were interviewed. Table 1 shows that the majority of participants were female (63.3%) with the mean age being 45.0 ± 14.9. The majority (58.9%) had partial or complete secondary schooling. Two-thirds of participants were unemployed, with most of those employed having only part-time work (26.7%). Computer ownership was low (4/90; 4.4%), whereas cellphone ownership was relatively high (67.8%) in this low-SES population. A third (33.3%) had smartphones; however, when asked about the ability to use their smartphones to look for health information, only 22.2% reported positively. A high of 91.1% reported being able to read in their home language (isiXhosa); this figure declined to 72.2% for English reading ability. Just over one-third reported having a chronic health condition.

**TABLE 1:** Participants’ sociodemographic characteristics (*N* = 90).

Variables	Frequency	%
**Gender**		
Male	33	36.7
Female	57	63.3
**Age (years)**		
18–29	15	16.7
30–44	30	33.3
45–59	28	31.1
60 and above	17	18.9
**Education (years)**		
0–7 (primary)	37	41.1
11–12 (secondary)	53	58.9
**Employed**		
Full-time	6	6.7
Part-time	24	26.7
**Computer**		
Own a computer	4	4.4
Can use a computer	29	32.2
Can look for information on a computer	22	24.4
**Cellphone ownership**		
Own a cellphone	61	67.8
**Smartphone ownership and use**		
Cellphone is a smartphone	30	33.3
Can use a smartphone to look for health information	20	22.2
**isiXhosa literacy**		
Understand and responds but cannot read	8	8.9
Understands, responds and can read	82	91.1
**English literacy**		
No understanding of English	7	7.8
Basic understanding but cannot respond	8	8.9
Understands and responds but cannot read	10	11.1
Understands, responds and can read	65	72.2
**Chronic condition**	34	37.8
**Health literacy**		
Inadequate (1–10)	28	31.1
Marginal (11–17)	48	53.3
Adequate (18–24)	14	15.6

Pictogram comprehension results from this study are displayed in Table 2. In addition, for comparative purposes only, nine comprehension results from the prior ‘2012 study’ and one result from the 2009 Ramela study are also displayed. However, it should be observed that no comparative statistical analysis could be performed as the criteria for inclusion, although conducted in the same Xhosa target population, were slightly different. The mean overall pictogram comprehension score was 7.9 ± 1.8 (maximum score of 10). The two most poorly comprehended pictograms were ‘general body pain’ (51.1%) and ‘dizziness’ (54.4%). The remaining eight pictograms all complied with the ISO criterion of 66.7% correct comprehension. Misinterpretations of individual pictograms are displayed in Table 2.

**TABLE 2:** Pictogram comprehension and misinterpretation (*N* = 90).

Pictogram comprehension		ISO compliant	Incorrect comments
General body pain 51.1% (2012: 52.5%)†	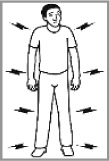	✘	Seems to be fine; not feeling well; can’t walk; is sick; stomach problems. Bolt symbol: arthritis; back pain; shoulder problem; sore arms; pain in joints; chest pain; body heating up; has fever; shivering and getting cold; has TB.
Constipation 80.0% (2012: 25.0%)†	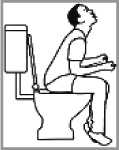	✔	Has diarrhoea and/or runny tummy; having seizures in toilet; stomach-ache; has nosebleed so is looking upwards; having a stroke; thinking and praying hard.
Diarrhea 94.4% (2009: 92.5%)‡	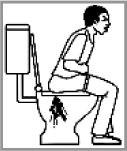	✔	On the toilet; having seizures on the toilet; stomach ache and is holding his stomach.
Cough 91.1% (2012: 47.5%)†	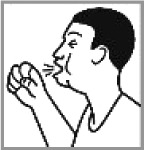	✔	Talking with breath coming out; holding an asthma pump; yawning; trying to move something out from stomach.
Dizziness 54.4% (2012: 20.0%)†	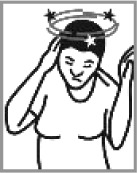	✘	Earache; itchy head; headache; cuts or sores on the head; nervous, worried, stressed, depressed or irritated; mental problems; high BP; thinking a lot; seeing stars; stars show it is a migraine; has a stroke.
Headache 93.3% (2012: 87.5%)†	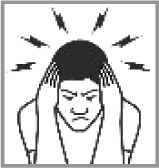	✔	Person thinking hard; painful ears; depression; head feeling hot.
Heartburn 71.1% (2012:45.0%)†	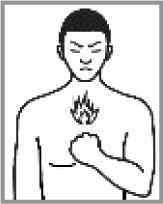	✔	Chest pain; heart problem; sore throat; coughing as chest is blocked; mucous in chest; ulcer Fire symbol: a sore; heart and/or lung problems; mucous in the chest; chest pain; sore throat; coughing as chest is blocked.
Rash 74.4%(2012: 87.5%)†	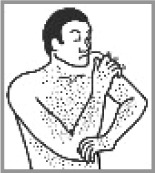	✔	Pain on shoulder; painful arm; shoulder pain and shingles; washing his body; holding his arm; body is sweating; fever; chicken pox; pimples.
Fever 85.6% (2012: 17.5%)†	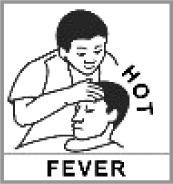	✔	Headache; being prayed for because of headache; nurse holding man’s head; doctor holding man’s head that is hot.
Vomiting 93.3%(2012: 87.5%)†	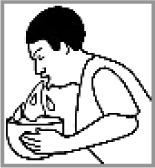	✔	Weak, coughing and spitting; drinking water from bowl; coughing out something.

Note: The nine results from 2012 and one from 2009 apply to older versions of the pictogram, not the version displayed in this table.

IOS, International Organization for Standardization.

† , Results presented in Dowse et al. (2022);

‡ , Result presented in Ramela (2009).

Health literacy testing revealed that the majority of participants were classified as having marginal HL (53.3%), with only 15.6% achieving adequate HL status. The mean HL score was 12.7 ± 4.3. In Table 3, the number and percentage responses for each pictogram correctly interpreted is displayed in the three HL categories. These are displayed in descending order of the total response.

**TABLE 3:** Correct pictogram comprehension per health literacy category.

Pictogram	Correct total response from highest to lowest							*p*
	Inadequate (*N* = 28)		Marginal (*N* = 48)		Adequate (*N* = 14)		Total	
	*n*	%	*n*	%	*n*	%		
Diarrhoea	24	85.7	47	97.9	14	100.0	85	0.06
Vomiting	24	66.7	46	33.3	14	100.0	84	0.19
Headache	25	89.3	45	93.8	14	100.0	84	0.61
Cough	23	82.1	45	93.8	14	100.0	82	0.14
Fever	20	71.4	43	89.6	14	100.0	77	0.03 *
Constipation	20	71.4	39	81.3	13	93.0	72	0.26
Rash	19	67.9	36	75.0	12	86.0	67	0.48
Heartburn	15	53.6	36	75.0	13	93.0	64	0.03 *
Dizziness	10	35.7	30	62.5	9	64.0	49	0.06
General body pain	17	60.7	20	41.7	9	64.3	46	0.16

* , Indicates significant difference in comprehension between categories.

In Table 4, categorical analysis of HL category and comprehension score indicated a significant association (*p* = 0.002), with the mean comprehension score increasing by approximately one point for each increasing HL category. Regression analysis showed a significant increase in comprehension with improvement in HL level (*p* = 0.001), although the beta coefficient of 0.14 reveals the slope of the line as being close to horizontal. Post hoc analysis indicated a significant difference in comprehension score between the ‘inadequate’ and both other HL groups (marginal and adequate HL). Correlation analysis revealed a moderate positive correlation of HL with comprehension score (*r* = 0.34, *p* = 0.001). Using Fisher’s exact test, differences in comprehension between the HL groups were only statistically significant for ‘heartburn’ and ‘fever’ (*p* = 0.03 for both). However, it is evident that those participants with adequate HL consistently achieved better comprehension and had the lowest rate of pictogram misinterpretation. Five of the 10 pictograms were correctly comprehended by all 14 participants who achieved adequate HL scores.

**TABLE 4:** Association between health literacy and pictogram comprehension score.

Comprehension score (mean ± s.d.)	Health literacy		*p*	Post hoc comparison
	Category	*n*		
7.04 ± 2.10	Inadequate (≤ 10)	28	0.002	Reference	
8.06 ± 1.62	Marginal (11–17)	48	-	0.013, Reference	
9.00 ± 0.88	Adequate (18–24)	14	-	< 0.001, 0.002	

Note: Beta coefficient = 0.14, *r* = 0.34, *p* = 0.001, 95% confidence interval: 0.06–0.22.

s.d., standard deviation.

As can be seen in Table 5, pictogram comprehension was significantly negatively associated with age (*p* < 0.006) although, from post hoc analysis, the only significant differences in comprehension between age groups occurred between the ≥ 60-year group with all three other age groups. Education was strongly associated with pictogram comprehension (*p* < 0.001) and indicated a significant increase in comprehension score from primary to secondary school. English proficiency was also associated with an increasing comprehension score (*p* < 0.001) with the lowest group (no understanding of English) having a significantly lower pictogram comprehension than for all other groups. The highest English proficiency group achieved the top comprehension score, although this score was not significantly different from the group who could understand and respond, but not read English.

**TABLE 5:** Impact of age, education and English proficiency on pictogram comprehension and health literacy.

Variable	*n*	Comprehension score (Max = 10)		Health literacy score (Max = 24)	
		Mean ± s.d.	*p*	Mean ± s.d.	*p*
**Age (years)**					
18–29	15	8.5 ± 1.5	0.006	13.5 ± 3.7	< 0.001
30–44	30	8.4 ± 1.7		15.2 ± 3.9	
45–59	28	7.7 ± 1.8		11.7 ± 3.7	
≥ 60	17	6.7 ± 1.8		9.3 ± 3.7	
**Education (years)**					
0–7 (primary)	37	6.9 ± 1.9	< 0.001	9.9 ± 3.5	< 0.001
8–12 (secondary)	53	8.5 ± 1.4		14.7 ± 3.7	
**English proficiency**					
No understanding	7	5.4 ± 2.2	< 0.001	10.0 ± 3.0	< 0.001
Understand, not respond	8	6.1 ± 1.8		8.3 ± 3.2	
Understand, respond	10	7.5 ± 2.0		9.9 ± 3.5	
Understand, respond, read	65	8.4 ± 1.4		14.0 ± 4.0	

s.d., standard deviation.

Similar trends were observed when investigating the impact of these three variables on the HL score (Table 5). Health literacy had an overall significant negative association with age (*p* < 0.001), although the increase in HL was not linear. Older participants (e" 60) had significantly lower HL than the other three age groups, whereas those in the age category 30–44 years had the highest HL score. A significant positive relationship (*p* < 0.001) with HL was established for both education and English proficiency. The highest English proficiency group achieved a significantly higher HL score than the other three groups (*p* < 0.001).

Every single participant responded positively to questions investigating their opinion of the pictograms. They were all enthusiastic about the suggestion that pictograms be used on medicine boxes or packets to assist recall of medicine indication and use. A few commented that this would be particularly useful when they had to take multiple medicines.

## Discussion

This study aimed to assess the association of HL with the ability to comprehend pictogram content. An overall significant, moderate association of HL with pictogram comprehension was found. A trend showing an increase in comprehension score from lower to higher literacy categories was identified. However, differences in mean comprehension score between the three categories were not significant, although misinterpretation was more common in the lower HL categories. This suggests that HL category alone should not be used as a reliable predictor of the ability to comprehend visual images.

There is a paucity of literature directly addressing the influence of different HL levels on the ability to interpret the meaning of the pictogram’s intended message. The literature does include descriptions of pictogram-based HL interventions, but in many cases HL is not assessed. In those studies where HL is assessed, the authors noticed no stated intention to explore the association of differing levels of HL with pictogram comprehension. This indicates that our article is well positioned to contribute unique data to the knowledge base of pictogram interpretation and its determinants.

However, in accordance with our findings, previous studies found that it was the low HL participants who experienced the most difficulties comprehending the pictogram meaning. A qualitative study by Wolpin et al. (2016) aimed to refine a set of pictographs to use as medication reminders. Participants were screened to ensure only those who met the criteria for low HL using the S-TOFHLA measure were recruited. The study concluded that low HL adults encountered difficulties comprehending many of the common pictograms used on the study medication labels. A study by Yin et al. (2011) aimed to determine whether a pictographic dosing diagram influenced the ability of caregivers to dose infant paracetamol. It also determined if different levels of HL, assessed using the Newest Vital Sign measure, influenced pictogram benefit. They concluded that the pictographic dosing diagram may assist caregivers, particularly those with low HL, to administer the correct dose.

Health literacy is considered to be a midstream determinant of health (Nutbeam & Lloyd 2021) with the potential to mediate the causes and effects of established social determinants of health. Lower HL is consistently associated with poor social and economic conditions (Stormacq, Van den Broucke & Wosinski 2019), reflecting the conditions that prevail in this study setting. The importance of the social context of HL for vulnerable groups such as LEP populations has been observed, and also applies to communities with a strong family or communal orientation (Fry-Bowers et al. 2014; Sentell & Braun 2012). As was evident in the population in this study, such communities often shape attitudes to and knowledge of various health conditions, can influence health behaviour and treatment, and communally participate in decision-making about health concerns (Marimwe & Dowse 2019).

The HELT-LL was designed for application in limited literacy populations and has a lower overall cognitive demand. It not only includes items requiring application of cognitive skills but also expands the focus to the broader social skills for dealing with and acting on health information that can influence overall HL. Despite the lower cognitive demand of this measure, only 15.6% of the study population achieved adequate HL status. It is noteworthy that all participants in the highest HL group correctly interpreted 5 out of 10 pictograms and demonstrated significantly fewer incorrect interpretations of the remaining five pictograms. This clearly identifies the presence of higher visual literacy skills in the higher literacy group, similar to other findings (Algabbani et al. 2022). Paradoxically, however, it should be observed that in our study a number of participants with inadequate HL achieved high pictogram comprehension scores, suggesting that factors other than good HL influence the ability to recognise and integrate elements in pictograms to uncover their meaning. Interestingly, Yin et al. (2011) also noticed an anomaly in their study of high error rates occurring even among caregivers with adequate HL. In accordance with previous findings, education level was identified as a determinant of successful pictogram comprehension (Dowse & Ehlers 2003; Merks et al. 2018). However, literacy is not a fixed asset but can be improved (Nutbeam & Lloyd 2021). Reporting the number of completed years within a formal schooling system is not necessarily an accurate reflection on current general literacy status, which may have been improved either formally through courses or informally.

Individuals with low HL and LEP constitute a vulnerable group with a higher risk of poor health status, disease management and health outcomes (Pandey et al. 2021; Sentell & Braun 2012; Stormacq et al. 2019). In this study, almost three quarters of the participants reported being able to understand, respond and read in English. However, a question in the HELT-LL formally establishes the degree of assistance required in reading health information by asking participants to read a short section of a patient medicines information leaflet. Less than a quarter of participants (22%) were able to successfully read and explain the information. In South Africa this information is presented in English and Afrikaans but, unfortunately, is not available in any of the local African languages. This highlights the limitations of these manufacturer-developed leaflets as being too complex with an inappropriately high readability level, placing high cognitive demands on the average South African patient (Krige & De Wet 2009). Simplifying the language in these leaflets and including well-designed and validated pictorial content could enhance readability of these leaflets (Browne et al. 2019).

The poorest performing pictogram in participants from all three HL groups was ‘general body pain’ which generated the highest number and diversity of incorrect interpretations. Difficulty with attempting to portray a generalised concept of pain as opposed to localised pain occurring in one clearly identified body site has been reported by others (Berthenet, Vaillancourt & Pouliot 2016), which suggest that the visual content of this pictogram should be reconceptualised to improve its comprehensibility. ‘Dizziness’, the second most poorly interpreted pictogram, has also been reported by other researchers as problematic (Berthenet et al. 2016). This pictogram included a ‘circles and stars’ visual above the head to suggest a dizzy state which, although familiar to many, is a graphic convention that requires active learning. Misinterpretation percentages were similarly high in the two lower HL groups. A follow-up study with the same participants to retest this pictogram after the meaning had been explained would have been valuable in establishing the extent of retention and recall of this challenging visual.

Misinterpretation of the ‘fever’ pictogram showed a clear progression from zero incorrect in the adequate HL group to significantly higher misinterpretation in the two lower HL groups. The pictogram included two words (‘hot’ and ‘fever’) that may not have been fully comprehended by these LEP participants, with meaning having to then be drawn from posture alone. This is reflected in comments such as the doctor and/or nurse was holding the man’s head and being prayed for because of a headache. Initial versions had also included the graphic convention of wavy lines to reflect heat radiation around the head area, but these were discarded after feedback from the pilot study. In retrospect, both versions (with and without heat lines) should have been tested for a more conclusive decision on the final version.

Access to digital technology is increasingly being recognised as a determinant of health, which would also impact HL (Rice & Sara 2019). Despite widespread mobile phone penetration in many LMICs, digital literacy barriers are still common among vulnerable populations (Nouri et al. 2019) concurring with our findings, which showed only around 25% reporting an ability to use computers or cellphones to look for health-related information. However, increasing desire in lower literacy populations to use this technology would enhance familiarity with its use and may mediate barriers to improving reading and the visual literacy skills required for comprehending pictograms, with a possible positive impact on HL.

Strengths of this study include the use of a locally validated HL test that was developed specifically for a population such as the one in this study. There are not many HL tests developed in, and for, LMICs and those with limited literacy. The pictograms used had previously undergone multiple ‘design-test-modify-test’ cycles and had therefore addressed a common criticism in the pictogram literature, which notes the generally poor quality of health and medicine-related pictograms. Limitations include the research being conducted at one study site in only one of the African language groups. Results are therefore not necessarily generalisable within South Africa or further afield. Three student researchers collected all data; although extensive training was offered and early interviews were conducted under the supervision of the experienced lead researcher, it is possible that minor differences in assessment of comprehension may have occurred. To mitigate this possibility, meetings were held to discuss possible uncertainties in scoring pictogram comprehension.

Future research should focus on vulnerable groups who are most likely to encounter problems with pictogram comprehension such as those with lower HL and LEP. Reviews of the literature have observed the poor quality of current health pictograms; future research should include a graphic designer and end-users in the design team and should aim to generate high quality pictograms by adopting a multistage design-test-modify-retest process. Existing interventions demonstrate the potential to improve HL among higher-risk populations (Nutbeam & Lloyd 2021); these HL interventions should include visual content of a high quality and focus on reaching and engaging those groups disproportionately affected by low HL.

## Conclusion

This study found a significant positive overall relationship between HL and pictogram comprehension. Participants with adequate HL displayed higher pictogram comprehension skills than those with lower HL, with five pictograms being correctly comprehended by all in this group. However, there was no clear differentiation of comprehension by HL status between the two lower HL groups. Therefore, knowledge of HL status could, for those with adequate HL, be a useful predictor of the ability to comprehend pictograms. However, potential comprehension problems should be anticipated for those individuals who demonstrate lower HL status. Low HL, LEP and low education levels should all be regarded as potential indicators for possible misinterpretation of all visual images, including pictograms.
